# Animal Models for the Study of Hepatitis B Virus Pathobiology and Immunity: Past, Present, and Future

**DOI:** 10.3389/fmicb.2021.715450

**Published:** 2021-07-16

**Authors:** Xiaonan Zhang, Xiaomeng Wang, Min Wu, Reena Ghildyal, Zhenghong Yuan

**Affiliations:** ^1^Centre for Research in Therapeutic Solutions, Faculty of Science and Technology, University of Canberra, Canberra, ACT, Australia; ^2^Shanghai Public Health Clinical Center, Fudan University, Shanghai, China; ^3^Key Laboratory of Medical Molecular Virology, School of Basic Medical Sciences, Shanghai Medical College, Fudan University, Shanghai, China

**Keywords:** HBV, animal model, CccDNA, chronic hepatitis B, humanized mice

## Abstract

Hepatitis B virus (HBV) infection is a global public health problem that plagues approximately 240 million people. Chronic hepatitis B (CHB) often leads to liver inflammation and aberrant repair which results in diseases ranging from liver fibrosis, cirrhosis, to hepatocellular carcinoma. Despite its narrow species tropism, researchers have established various *in vivo* models for HBV or its related viruses which have provided a wealth of knowledge on viral lifecycle, pathogenesis, and immunity. Here we briefly revisit over five decades of endeavor in animal model development for HBV and summarize their advantages and limitations. We also suggest directions for further improvements that are crucial for elucidation of the viral immune-evasion strategies and for development of novel therapeutics for a functional cure.

## Introduction

Over half a century has passed since a precipitin line in an immunodiffusion agar gel was formed between serum of a hemophilia patient receiving transfusion and serum of an Australian aborigine (Blumberg et al., 1965). This serendipitous event turned out to be the first revelation of a new etiology: Hepatitis B Virus (HBV) (Sutnick et al., 1969; Dane et al., 1970; Almeida et al., 1971). The active research surrounding this virus thereafter has yielded in-depth understanding of its natural history, immunobiology, and pathogenesis.

HBV belongs to the *hepadnaviridae* which is characterized by a small genome (3.2 kilobase) of partially double-stranded DNA. It enters the human hepatocytes via the interaction between viral envelope protein and human sodium taurocholate co-transporting polypeptide (NTCP) in a highly species-specific manner (Yan et al., 2012; He et al., 2015; Burwitz et al., 2017; Takeuchi et al., 2019; Chen et al., 2020). It then reproduces its genome by a transcription—reverse transcription process. The former is done under the genetic instruction of the covalently closed circular DNA (cccDNA) which is formed by repairing the incoming viral genome or recycling of the viral DNA produced in the cytoplasm. The cccDNA serves as the template for transcription of the pregenomic RNA and other mRNAs that encode at least seven proteins. The pregenomic RNA is not only the messenger for viral core and polymerase, but also the template for reverse transcription, which strictly requires polymerase assisted core particle formation. HBV is a hepatotropic virus with strict host and organ tropism.

Individuals chronically infected with HBV can develop a series of liver diseases, from liver fibrosis, cirrhosis to hepatocellular carcinoma. Although great progress has been made in the prevention and treatment of chronic hepatitis B (CHB), there are still over 240 million people infected with HBV and around 650,000 people die of this disease each year globally (World Health Organization [WHO], 2015). To further reduce these figures, besides broader coverage of HBV vaccination, novel approaches of therapy that are able to cure chronic infection are needed. The continuing development of animal models for HBV infection has been instrumental for our understanding of its lifecycle, development of antiviral agents, and testing of preventive measures. A series of models have been developed ever since the “Australian antigen” was discovered. Their utilities and shortcomings are reviewed (Table 1). Possible directions for further development to meet the needs of an HBV cure are also discussed.

**TABLE 1:** Basic features of animal models for major hepadnaviruses.

Animal	Virus/vector	Virological characteristics			Immune response			Pathogenesis			Therapy	
					
		Viral entry	cccDNA formation	Persistence	Immune competence	T cell response	(HBsAg) B cell response	Inflammation	Fibrosis	HCC	Direct Antiviral	Therapeutic vaccine
Chimpanzee	HBV	+	+	age and dose-dependent	+	+	+	+	−	−	+	Yes but limited
Tree shrew	HBV	+	+	age-dependent	+	+	+	+	+	+	+	Yes but limited
Duck	DHBV	+	+	age and dose-dependent	+	+	+	+/−	−	−	+	Not relevant
Woodchuck	WHV	+	+	age-dependent	+	+	+	+	+	+	+	Yes
Mouse	Transgenic mice	−	−	Integrate	tolerance	−/+^c^	−/+^c^	−	−	+/−^e^	+	Yes^c^
	Hydrodynamic injection	−	−	Pro-clearance	+	High	High	−	−	−	+	Yes
	Adv vector	−	−/+^a^	Dose-dependent^b^	+	Medium	Medium	−	−	−	+	Yes
	AAV vector	−	−/+^a^	Pro-persistence	+	low	low	−	−	−/+^f^	+	Yes
	Mice with humanized liver	+	+	persistence	−	−	−	+	−	−	+	Inapplicable
	Dual humanized mice	+	+	Pro-persistence	+	+	±^d^	+	±^d^	−	+	Not studied

*^a^cccDNA formation can be achieved in certain designs such as using recombinant cccDNA techniques (refs. Qi et al., 2014; Yan et al., 2017). ^b^Adenoviral vector mediated HBV replication mostly cause transient viremia although higher persistence rate can be achieved with lower inoculum (ref. Huang et al., 2012). ^c^HBV transgenic mice are usually tolerant to constitutively expressed viral antigens. However, some reports show the activity of virus specific T and B cell responses (refs. Fumagalli et al., 2020; Michler et al., 2020). ^d^Depends on the type of dual humanized mice. ^e^HCC formation is observed in some transgenic lines. ^f^ HCC formation is observed in one report.*

## Chimpanzee and Other Primates

Apart from human subjects, chimpanzees were almost the only model used in the early stages of HBV research (McAuliffe et al., 1980). In as early as 1969, two groups independently reported the appearance of Australian antigen and development of its antibody after inoculation of positive sera in chimpanzees (Hirschman et al., 1969; Lichter, 1969). This was followed by several other studies with more detailed longitudinal observations (Maynard et al., 1971, 1972; Barker et al., 1973). Chimpanzees were also the only feasible subjects for evaluation of the first generation vaccines which were purified from HBV positive sera and inactivated (McAuliffe et al., 1980). A major achievement of molecular virology in 1982 was also made possible by injecting cloned viral genome into chimpanzees thus establishing an acute viral hepatitis (Will et al., 1982). All these studies established the molecular etiology of HBV as the causative agent for hepatitis B.

The fact that chimpanzees have the genetic background and immune system that are closest to humans makes them the host of choice for studying the innate and adaptive immune response to HBV infection. One striking feature of HBV acute infection observed in chimpanzees is that the virus does not induce or repress host gene expression in the lag (week 0–2) and logarithmic expansion phase (week 4–6) which is in stark contrast to HCV infection (Wieland S. et al., 2004). The induction of interferon stimulated genes (ISGs) and other inflammatory genes are within the viral clearance phase (week 8–12) which is initiated by infiltration of T cells, B cell, macrophages, and NK cells, etc. Transient antibody-mediated depletion of CD8 + T cells during the logarithmic phase of viral replication caused prolonged viremia and liver damage (Thimme et al., 2003). Meanwhile, the essentiality of the priming of CD4 + T cells in the early phase of the infection was also discovered, as pre-depletion of these cells caused persistent infection with minimal immunopathology (Asabe et al., 2009) reminiscent of the immune tolerance state of CHB. Most interestingly, inoculation of chimpanzees with intermediate viral dose (10^4^ to 10^7^ genome copy) leads to viral clearance whereas high-dose (10^10^ genome copy) or low-dose (10 copy) leads to persistent infection, indicating that kinetics of viral spread in the early phase determines the fate of the disease (Asabe et al., 2009). In a self-limited infection, an early (week 8–12) non-cytolytic suppression of viremia and a late (week 14–20) cytolytic destruction of HBV positive hepatocytes were both documented by longitudinal observations in chimpanzees, suggestive of a two-phase dynamic process (Guidotti et al., 1999; Wieland S.F. et al., 2004).

The use of chimpanzees has also facilitated the development of novel therapeutics. GS-9620 is a potent, orally active TLR7 agonist in clinical development for treatment of CHB. Its short-term oral administration in HBV infected chimpanzees achieved long-term suppression of viral load by inducing ISGs and cytokines/chemokines (Lanford et al., 2013). Further transcriptomic and histological analyses revealed intrahepatic aggregates comprised of CD8 + T cells and B cells in the portal regions (Li L. et al., 2018). Therapeutic vaccination schemes (Pancholi et al., 2001) and anti-HBV monoclonal antibodies (Eren et al., 2000) were also evaluated in chimpanzees preclinically. Unfortunately, due to increasing concerns over animal ethics, experiments using chimpanzees have been highly restricted (Altevogt et al., 2012).

Apart from chimpanzees, macaques were also suggested to be susceptible to HBV. Inoculation of HBV replicative plasmid into macaques (*Macaca Silvanus)* caused viremia and pathological changes (Gheit et al., 2002). The same group later found *Macaca fasicularis* from Mauritius island had a high positive rate of HBV DNA although at low viral load (Dupinay et al., 2013). Genome sequencing revealed that it was a genotype D subtype ayw3 with a substitution at position 67 within preS1. Inoculation of virus-positive pooled serum into *Macaca fasicularis* caused an acute infection. However, another study failed to establish an infection using the virus harboring this variation and using the same species of macaque (Burwitz et al., 2017). Some other HBV-related viruses were also identified in New World monkeys. The woolly monkey hepatitis B virus (WMHBV) infects its natural host, *Lagothrix lagotricha* (woolly monkey) (Lanford et al., 1998). Spider monkeys and chimpanzees were also shown to be susceptible to WMHBV in experimental infections (Lanford et al., 1998, 2003). The capuchin monkey hepatitis B virus (CMHBV) was recently identified in *Sapajus xanthosternos* in Brazil (de Carvalho Dominguez Souza et al., 2018). The surface protein of these two viruses showed high antigenic relatedness as evidenced by cross-reactivity of polyclonal antibody against HBV surface antigen. Furthermore, Hepatitis D Virus pseudotyped with WMHBV and CMHBV surface proteins could infect human hepatocytes suggesting their highly similar cellular entry process. Indeed, molecular substitution assays on key residues on the NTCP polypeptide suggested that amino acid 158 is critical for virus entry (Takeuchi et al., 2019). This residue in New-World monkeys, which include capuchin monkey and woolly monkey, is identical to that of human and chimpanzee. However, these animals not readily available as experimental hosts due to economical and ethical reasons.

## Tree Shrew

Tree shrews (*Tupaia belangeri*) are small mammals closely related to primates. The susceptibility of tree shrews to HBV infection was confirmed both *in vivo* and *ex vivo* (Walter et al., 1996; Yan et al., 1996) although a transient and low level of viremia was documented in newborns (Walter et al., 1996). A larger scale, longitudinal research on 46 tree shrews neonatally inoculated with HBV resulted in 6 chronic infections (Wang et al., 2012). Hepatic histopathological changes observed in chronically infected animals were similar to those observed in CHB (Ruan et al., 2013). Continued observation showed that hepatocellular carcinoma occurred in two of the six animals at the late stage of life (Yang et al., 2015). Thus, this model faithfully recapitulates many aspects of the HBV infection in humans. Another contribution made by this model is the study of the viral entry route. Fine mapping of the receptor binding site of PreS1 was made possible using primary hepatocytes isolated from *Tupaia* (Glebe et al., 2003; Glebe et al., 2005). More importantly, sodium taurocholate co-transporting polypeptide (NTCP) was identified as the functional receptor for HBV using primary culture of *Tupaia* hepatocytes (Yan et al., 2012).

## The Woodchuck and Duck Models

The Woodchuck Hepatitis Virus (WHV) (Summers et al., 1978) was first discovered in the late 1970s. It has significant similarities to HBV in its morphology, genome structure, and replication scheme. Its genome is frequently integrated into the host genome at the N-myc loci (Fourel et al., 1990) and is directly linked with the development of hepatocellular carcinoma (Gouillat et al., 1997). More detailed analysis on HBV showed that integration events are randomly distributed among chromosomes (Mason et al., 2016), the enriched loci found in human liver tumors such as TERT, MLL4, and CCNE1 are the result of clonal expansion (Sung et al., 2012; Zhao et al., 2016). Molecular virology studies revealed that various forms of linear viral DNA can be produced during WHV replication which serves as the substrate for chromosomal integration (Yang et al., 1996). These forms of viral DNA and its integration was also confirmed in CHB (Tu et al., 2021). Woodchuck has also been widely used for the evaluation of therapeutic solutions. The administration of nucleoside analogs on WHV infected animals inhibited viremia but did not inhibit its genetic reservoir (cccDNA) (Moraleda et al., 1997; Dandri et al., 2000). Woodchucks were also used as a testing ground for various combination therapies involving DNA vaccine, antigen-antibody immune complex, immune checkpoint modulators etc. (Roggendorf et al., 2007; Lu et al., 2008; Kosinska et al., 2012, 2013; Liu et al., 2014) with considerable success.

The duck hepatitis B virus (DHBV) (Mason et al., 1980) was first reported to be present in Pekin ducks in 1980 which bears significant resemblance to HBV in its genome, morphology, and replication. Because ducks are much more accessible than other hosts, DHBV was used as a model system for studying the replication scheme of hepadnaviruses. Indeed, Summers and Mason found that DHBV replicates its genome by reverse transcription of an RNA intermediate, i.e., the pregenomic RNA (Summers and Mason, 1982) thus pointing to its evolutionary kinship with retroviruses (Miller and Robinson, 1986). Moreover, *ex vivo* infection of primary duck hepatocytes revealed the existence of a nuclear cccDNA pool which is not maintained by semiconservative replication but by intracellular recycling of relaxed circular DNA (Tuttleman et al., 1986). The abundance of cccDNA is regulated by the expression level of viral surface protein which determines the relative rate of viral release and intracellular recycling of relaxed circular DNA (Summers et al., 1990). This phenomenon is also corroborated in HBV infection (Lentz and Loeb, 2011; Zhang et al., 2016). By biochemical purification, researchers identified the receptor for DHBV as a 180 kDa glycoprotein (Kuroki et al., 1994; Breiner et al., 1998; Urban et al., 1998), although the entry of HBV does not use human homologs of this protein.

The above-mentioned animal models can be naturally infected with members of hepadnaviruses. There are, however, quite several limitations that restrict further studies on HBV pathogenesis, immune response, and curative strategies. The Pekin duck is a powerful tool for DHBV molecular virology, but its immune system is evolutionarily distant from that of humans. The limited supply of woodchucks and the difficulty in their domestication restrict their larger-scale experimentation. The chimpanzees have been historically crucial for the development of prophylactic vaccine and for studying HBV immunobiology, but their high cost, genetic variations, and ethical regulations have prohibited their further use. Experimental mice have long been the model of choice in immunologic and pathological studies, but they are not susceptible to HBV or other hepadnaviruses. A large number of studies have been performed to establish mouse systems that can address specific aspects of HBV biology.

## Transgenic Mice

With the development of transgenic mice technology, researchers started to clone HBV sequences into the mouse germline in the 1980s. The resultant mouse strains can be used to study the pathophysiological effects of certain viral gene products. For example, transgenic expression of surface antigen does not cause obvious pathologies (Babinet et al., 1985; Chisari et al., 1985), but the enforced expression of the large surface protein inhibits the release of small surface protein (Chisari et al., 1986), increases proteinaceous cytoplasm containing acidophilic inclusions resembling ground-glass hepatocytes observed in human carriers of HBV, and later develop nodular hyperplasia (Chisari et al., 1987). The oncogenic role of HBx is established by studies on transgenic mice. Kim et al. developed a transgenic mouse line in which HBx was expressed under the control of its native promoter (Kim et al., 1991). Histopathology begins with multifocal preneoplastic lesions followed by benign adenomas and finally malignant carcinomas. They also found that male mice developed disease and died much earlier than females. A similar observation was made in an HBx transgenic mouse lineage with high level of protein expression (Koike et al., 1994). Apart from the reported findings, they suggested that the formation of full-blown carcinoma requires additional genetic events as only small populations of altered foci developed into neoplasia. Indeed, they observed increased DNA synthesis and aneuploid DNA content in a subset of hepatocytes. Although there were a few reports that did not find significant histological alteration in HBx transgenic mice (Lee et al., 1990), the majority of them supported its tumorigenic role (Tsuge et al., 2010; Quetier et al., 2015).

Thanks to the well-developed tools for immunologic studies, HBV transgenic mice were extensively used to elucidate the mechanism of immunopathogenesis and mechanism of virus-mediated tolerization. The knowledge on the functional role of HBeAg has been greatly expanded using e antigen transgenic mice. HBeAg is translated from a precore mRNA a few nucleotides upstream of the initiation codon of the core open reading frame and further processed by proteolytic cleavage. The primary amino acid sequence of HBeAg shows significant identity to that of HBcAg although HBeAg is structurally distinct and is expressed in non-particulate form and secreted into circulation. There are a large number of reports supporting the link between HBeAg and active manipulation of the anti-HBV immune response. For example, HBV G1896A mutation that causes the premature termination of the e antigen is associated with acute fulminant or severe hepatitis (Tong et al., 2013). Using HBeAg-expressing transgenic mice, Milich et al. (1990) found that these mice are immunologically tolerant to not only HBeAg but also HBcAg at the T-cell level. T cells exposed to HBeAg were non-responsive to HBcAg. In addition, after priming with core antigen, *in vitro* anti-HBc IgG production was greatly reduced in e antigen transgenic mice but IgM production was unaffected suggestive of a reduced Th-cell function. Moreover, non-transgenic mice exposed to HBeAg *in utero* by their transgenic mother showed reduced T cell response to HBcAg peptides which suggested that HBeAg can pass the placenta to induce fetal tolerance to HBcAg before and around birth. Thus, HBeAg plays a crucial role in restricting T cell response to HBcAg in the early phase of HBV infection and contributes to the establishment of chronic infection.

The transgenic mice expressing individual viral proteins are still limited by the lack of replicative parameters. The establishment of replication-competent HBV transgenic mice has provided a robust system for dissecting the innate and adaptive immune response within the context of persistent HBV infection (Guidotti et al., 1995). In this model, a high level of viral RNA and replicative intermediates are detected in the hepatocytes, and virus particles in serum are morphologically indistinguishable from those in natural infection. Although the host is immune tolerant to the virus, an acute immune response can be initiated by adoptive transfer of specific immune cells. Introduction of HBsAg-specific cytotoxic T lymphocytes (CTLs) in this transgenic mouse results in a transient spike of liver damage (Guidotti et al., 1996) whereby a small fraction of the hepatocytes are killed. Nevertheless, these CTLs clear all traces of HBV gene expression and replication via the antiviral activity of IFN-γ and TNF-α. Indeed, the potent suppression of viral replication is independent of the cytolytic activity as perforin knockout CTLs also abolish viral activity. Nevertheless, the initial cell lysis triggers focal inflammation response in which antigen-non-specific cells such as neutrophils, polymorphonuclear neutrophils, and platelets infiltrate into the hepatic sites (Sitia et al., 2002, 2004; Iannacone et al., 2005). The polymorphonuclear neutrophils are known to express matrix metalloproteinases which facilitate the recruitment of mononuclear cells into the liver and exacerbate liver inflammation (Sitia et al., 2004). In addition to studies on CTL-mediated adaptive immune response, innate immune responses can also be investigated. Kakimi et al. analyzed the antiviral effect of activated natural killer T (NKT) cells by injection of antibody to galactosylceramide (Kakimi et al., 2000). Similarly, the functional role of Interleukin-12 (IL-12) (Cavanaugh et al., 1997), IL-18 (Kimura et al., 2002), was also analyzed. This model is also suitable for evaluation of various antiviral therapies (Julander et al., 2002; Weber et al., 2002; Julander et al., 2003; Uprichard et al., 2005; Ebert et al., 2011; Buchmann et al., 2013).

It is also worth noting that although transgenic mice are thought to be completely tolerant to the transgene-encoded viral protein, spontaneous clearance of HBsAg due to the emergence of antibodies was reported (Fumagalli et al., 2020). Also, a therapeutic vaccination scheme combined with siRNA-mediated knockdown in HBV transgenic mice generated antibodies toward HBsAg (Michler et al., 2020). These results indicate that the transgene-specific B cells are not clonally deleted and may be activated in certain circumstances.

## Transfected or Transduced Mice

Although adoptive transfer experiments in HBV transgenic mice can inform the antiviral capability of various cell types, this information does not necessarily translate to a real infection as the cell type under question can be very scarce or functionally tolerized *in vivo*. Since mouse hepatocytes are not permissive to HBV even after the introduction of the human NTCP gene (He et al., 2015), methods that can introduce viral DNA without germline insertion are needed. The hydrodynamic injection method, which involves rapid, high-pressure tail vein injection of HBV constructs was developed. Yang et al. introduced an overlength replication-competent HBV genome into mice and observed antigen production, viral transcripts, and DNA synthesis within two weeks in wildtype and much longer in NOD/SCID mice (Yang et al., 2002). By optimization of the delivery construct, Huang et al. achieved over 6 months of antigenemia in 40% of the injected immunocompetent mice (Huang et al., 2006). Further studies demonstrated that mouse genetic background has a significant impact on the rate of clearance. BALB/c and NOD/ShiLtJ mice quickly cleared the virus while C3H/HeN mice are more tolerant to viral replication (Chou et al., 2015; Peng et al., 2015). An association with the gut microbiota and age-related viral clearance is proposed (Chou et al., 2015).

Mouse hepatocytes can also be transduced by recombinant viral vectors, e.g., adenovirus (Adv) or adeno-associated virus (AAV), to initiate HBV replication. Transduction by Adv-HBV usually results in a transient antigenemia (Sprinzl et al., 2001; von Freyend et al., 2011) although it can be prolonged by a lower dose inoculation (Huang et al., 2012). By contrast, the introduction of HBV genome via AAV results in an immunotolerant phenotype with long-term antigenemia and minimal liver inflammation and fibrosis (Yang et al., 2014). The strikingly different behavior of these two viral vector systems is thought to derive from the feature of the carriers, the former being an activator of innate immune response and the latter usually stealthy and silent (Greber and Flatt, 2019; Ertl, 2021).

A more recent development in this transient transfer method involves the formation of *bona fide* cccDNA within the nuclei which may better recapitulate the epigenetic state of the supercoiled episomal cccDNA in natural infection. To achieve this, different approaches were used. Yan et al. utilized a phage φC31 integrase-mediated intramolecular recombination technology to generate a recombinant cccDNA in *Escherichia coli* (Yan et al., 2017). The resulting DNA molecule is highly similar to the native cccDNA and can initiate viral replication and antigen expression within the mouse liver. Another approach used by Deng’s group utilized the Cre-loxP *in vivo* recombination system in which the vector backbone was excised by the co-introduced Cre recombinase. The remaining loxP site within the recombinant cccDNA (rcccDNA) is flanked by a pair of splice donor and acceptor sites. Thus, its transcript undergoes a post-transcriptional RNA splicing yielding an RNA pregenome identical to that of a wildtype virus (Qi et al., 2014). Hydrodynamic injection of this vector resulted in the formation of rcccDNA within the nuclei. The same group further improved this system by using a replication-defective adenoviral vector to transfer the rcccDNA into *Alb-Cre* transgenic mice which led to prominent HBV persistence for over 62 weeks (Li G. et al., 2018). We have also established an rcccDNA model based on the Cre-loxP recombination strategy by using a AAV8 vector that is hepatotropic and achieved long-term antigenemia and cccDNA persistence (Wu et al., 2020).

## Humanized Chimeric Mice

Although the HBV transgenic and transfected mice each partially recapitulate some features of CHB, neither of them supports the complete infection cycle of infection due to the lack of cellular receptor. Engraftment of susceptible human hepatocytes into mouse can overcome this barrier. The “trimera” mouse is the result of the earliest attempts in this direction. Using mice that lack mature T, B and NK cells, human liver fragments were transplanted under the kidney capsule. After injection of HBV positive sera, viral DNA and antigens were detected for about two months (Ilan et al., 1999; Eren et al., 2000). Although this model was used for evaluation of antiviral therapy and monoclonal antibodies against HBsAg, the short time window for infection and transient viremia offered limited operational capability. Another easy-to-use mouse model, in which the HBV replication-competent HepAD38 cells were subcutaneously engrafted into nude mice, may serve as a simple platform for antiviral evaluation (Feitelson et al., 2007; Schinazi et al., 2012). It was not until mouse strains that support expansion of human hepatocytes, that humanized chimeric mice became a robust infection model.

In the early 1990s, researchers found that mice with the liver specific expression of the urokinase-type plasminogen activator (Alb-uPA) resulted in elevated uPA concentration, hypofibrinogenemia and neonatal hemorrhaging (Sandgren et al., 1991). This feature was exploited to facilitate the repopulation of xenogenic hepatocytes in various genetic backgrounds of immune deficiency (Rhim et al., 1995; Petersen et al., 1998; Tateno et al., 2004). Dandri et al. established active HBV replication in uPA/RAG-2 mouse repopulated with human hepatocytes although the humanized efficiency is less than 15% (Dandri et al., 2001). Later development of uPA-SCID mice resulted in a more pronounced engraftment (Meuleman et al., 2005). Indeed, a high (10^10^ copies/ml) and long-lasting viral titre could be achieved (Tsuge et al., 2005). Now the uPA-SCID model has been widely utilized for evaluation of antivirals (Oehler et al., 2014; Mueller et al., 2018), engineered immune therapy (Koh et al., 2018), and mechanism of innate immune response against HBV (Lütgehetmann et al., 2011; Belloni et al., 2012).

A major drawback of the uPA-SCID model is that the uncontrolled expression of the uPA gene in very early life requires human hepatocytes xenograft soon after birth, which is difficult to manipulate and easily causes severe hemorrhage during operation (Meuleman and Leroux-Roels, 2008). The later development of the Fah (Fumaryl acetoacetate hydrolase) knockout mice largely resolved this issue. Fah is an enzyme that catalyze the last step of Tyrosine/Phenylalanine catabolism. The loss of Fah gene causes the accumulation of toxic metabolic intermediates in this pathway. Supplementation of 2-(2-nitro-4-fluoromethylbenzoyl)-1,3-cyclohexanedione (NTBC), an inhibitor that blocks the enzymatic conversion of upstream intermediate, to the drinking water prevents the liver toxicity (Grompe et al., 1995). This feature provides much more control over the extent of mouse liver damage and the timing of hepatocyte xenograft. Based on this, several humanized models were established (Azuma et al., 2007; Bissig et al., 2007), among which the Fah−/− mice with additional immune deficiency (Rag2−/− and IL-2rγ−/−), hence named FRG triple KO mice achieved the highest engraftment rate (90%) (Azuma et al., 2007). FRG KO mice have been successfully used to establish HBV and HCV infection (Bissig et al., 2010) and to evaluate the antiviral action of interferon-α subtypes (Chen et al., 2021).

A major limitation of the humanized mice described above is the lack of the adaptive immune system that can recognize the incoming HBV and make responses that contribute to viral clearance or immunopathology. To solve this, an A2/NGS-hu-HSC/Hep mice model was established (Bility et al., 2014). In this model, the human HLA-A2 allel was introduced into NOD/SCID/IL-2Rγ−/− background and transplanted with human CD34^+^ cells from fetal liver which serve as hematopoietic stem cells and liver progenitor cells for the reconstituted mice (Bility et al., 2014). Injection of anti-Fas agonistic antibody (Jo2) causes mouse hepatocyte damage and makes room for the introduced progenitor cells to settle in the liver. The human HLA-A2 transgene is thought to promote the HLA-restricted human T cell function. This model achieved around 25% repopulation of human hepatocytes and successful infection of HBV although with relatively low viral load (< 5 × 10^5^ copies/ml). Importantly, HBV specific CD8^+^ T cell response was elicited, and significant liver inflammation and fibrosis was observed. Notably, the authors observed intrahepatic accumulation of M2-like macrophage which is associated with accelerated liver fibrosis in CHB patients. The authors did not report humoral immune response against HBV. In another study, researchers developed the HIS-HUHEP model, in which CD34^+^ fetal derived human hematopoietic stem cells (HIS), and adult human hepatocytes (HUHEP) were introduced into a BALB/c Rag2/IL2rγ KO NOD, sirpa, uPA transgenic mice (Dusseaux et al., 2017). The introduction of adult hepatocytes caused much higher HBV viral load and antigens. Inflammatory cell infiltration surrounding core antigen positive hepatocytes was observed although no sign of fibrosis was found. The infiltrating lymphocytes included NK cells, CD4+ and CD8+ T cells with activation/exhaustion markers such as PD-1 and CTLA-4. Importantly, antibodies to surface and core antigens were detected. As the access to fetal hematopoietic stem cells and primary hepatocytes became more limited, other sources of stem cells were explored. Yuan et al. used human bone mesenchymal stem cells to repopulate in FRG mice in BALB/c SCID background (Yuan et al., 2019). Depletion of mouse hepatocytes was achieved by anti-Fas agonistic antibody (Jo2) combined with withdrawal of NTBC. Elimination of murine immune cells was secured by the injection of busulfan. This resulted in high liver chimerism and well differentiated hepatocytes. Reconstitution of the human immune cells was also evident with major myeloid and lymphoid cells. Persistent HBV infection with high viral load and antigen titers was observed. A unique feature of this model is that a large number of lymphocytes and macrophages infiltrated the liver after HBV infection, which was accompanied by high level of cytokines and chemokines. This caused progressive liver fibrosis and cirrhosis although viral loads and antigens were not significantly suppressed. Evaluation of the total serum human IgG and IgM revealed significant elevation during infection whereas antibodies to HBV antigens reached a peak at about 12 weeks post infection and then declined. It is possible that viral infection triggered an inflammatory milieu that fostered a polyreactive humoral antibody response in addition to viral antigens. Nevertheless, this dual humanized model constitutes a robust and easy-to-implement system for HBV pathobiology.

## Future Directions

With the help of *in vivo* models, remarkable achievements have been made in the inquiry into how HBV exploits the host molecular and cellular machineries to propagate, and in the development effective preventative and therapeutic measures to contain the spread and progression of this disease. The initial use of chimpanzees as the experimental host established the transmissibility of the “Australian antigen” and unraveled many key features of HBV induced immunologic responses. Studies using woodchuck and pekin duck infected with *Hepadnaviridae* family members elucidated the framework of viral life cycle and provided surrogate models for antiviral assessment. The endeavor to establish mouse models that can recapitulate different aspects of HBV-mediated disease has also yielded substantial progress.

With the even higher coverage of preventive vaccines and availability of antiviral therapies, the development of a therapeutic scheme that effectively reverses virus-mediated immunotolerance and establishes an immunodominance over HBV without triggering overt liver damage becomes the greatest challenge of our times. Such endeavor will require an immunocompetent small animal model that accommodates most steps of the viral lifecycle and is easy-to-manipulate genetically and immunologically. Obviously, none of the current models fully meet these requirements. The HBV transgenic mouse model is overly tolerant toward the virus while the hydrodynamic injection and Adenoviral transfer model are generally prone to resolution. The AAV model mostly generates a chronic infection reminiscent of a carrier state which seems to be a suitable system for evaluation of therapeutic vaccines. However, it remains to be shown that such an immunotolerant phenotype is characteristic of HBV itself but not of the vector. The dually humanized mice model is regarded as a promising direction. Various immunopathology of CHB, such as inflammation, liver fibrosis and cirrhosis have been recreated by the latest models. However, the engraftment of xenogenic liver and hematopoietic system causes mismatches in the HLA system between the xenograft and the host. Although graft-versus-host disease is not found in the reported systems (Dusseaux et al., 2017; Yuan et al., 2019), there are still concerns over whether the nature of immune recognition and reactions against the virus in such a system is in line with that of natural infection. This will directly affect their suitability in studying the immunologic determinants of viral tolerance and clearance. Indeed, disruptive innovations are required to remove these obstacles before mechanistic details of virus-mediated immunotolerance can be fully unveiled and therapies for a cure can be made.

## Author Contributions

XZ and XW conceived and drafted the manuscript. MW, RG, and ZY provided additional content to the manuscript and critically reviewed the text. All the authors read through the manuscript and agreed on the final test.

## Conflict of Interest

The authors declare that the research was conducted in the absence of any commercial or financial relationships that could be construed as a potential conflict of interest.
